# Overdose Deaths Related to Fentanyl and Its Analogs — Ohio, January–February 2017

**DOI:** 10.15585/mmwr.mm6634a3

**Published:** 2017-09-01

**Authors:** Raminta Daniulaityte, Matthew P. Juhascik, Kraig E. Strayer, Ioana E. Sizemore, Kent E. Harshbarger, Heather M. Antonides, Robert R. Carlson

**Affiliations:** ^1^Center for Interventions, Treatment, and Addictions Research, Department of Population and Public Health Sciences, Boonshoft School of Medicine, Wright State University, Kettering, Ohio; ^2^Montgomery County Coroner’s Office/Miami Valley Regional Crime Lab, Dayton, Ohio; ^3^Department of Chemistry, Wright State University, Dayton, Ohio.

Ohio is experiencing unprecedented loss of life caused by unintentional drug overdoses (*1*), with illicitly manufactured fentanyl (IMF) emerging as a significant threat to public health (*2*,*3*). IMF is structurally similar to pharmaceutical fentanyl, but is produced in clandestine laboratories and includes fentanyl analogs that display wide variability in potency (*2*); variations in chemical composition of these drugs make detection more difficult. During 2010–2015, unintentional drug overdose deaths in Ohio increased 98%, from 1,544 to 3,050.* In Montgomery County (county seat: Dayton), one of the epicenters of the opioid epidemic in the state, unintentional drug overdose deaths increased 40% in 1 year, from 249 in 2015 to 349 in 2016 (estimated unadjusted mortality rate = 57.7 per 100,000) (*4*). IMFs have not been part of routine toxicology testing at the coroner’s offices and other types of medical and criminal justice settings across the country (*2*,*3*). Thus, data on IMF test results in the current outbreak have been limited. The Wright State University and the Montgomery County Coroner’s Office/Miami Valley Regional Crime Laboratory (MCCO/MVRCL) collaborated on a National Institutes of Health study of fentanyl analogs and metabolites and other drugs identified in 281 unintentional overdose fatalities in 24 Ohio counties during January–February 2017. Approximately 90% of all decedents tested positive for fentanyl, 48% for acryl fentanyl, 31% for furanyl fentanyl, and 8% for carfentanil. Pharmaceutical opioids were identified in 23% of cases, and heroin in 6%, with higher proportions of heroin-related deaths in Appalachian counties. The majority of decedents tested positive for more than one type of fentanyl. Evidence suggests the growing role of IMFs, and the declining presence of heroin and pharmaceutical opioids in unintentional overdose fatalities, compared with 2014–2016 data from Ohio and other states (*3*–*5*). There is a need to include testing for IMFs as part of standard toxicology panels for biological specimens used in the medical, substance abuse treatment, and criminal justice settings.

The MCCO Toxicology laboratory provides postmortem forensic toxicology services to approximately 30 of Ohio’s 88 counties. Data from 281 unintentional overdose fatalities that occurred in Montgomery County and 23 additional counties^†^ during January and February 2017, were analyzed by the MCCO Toxicology laboratory, and had assigned causes of death as of May 8, 2017, were included in this study. Montgomery County data include all unintentional drug overdose deaths that occurred in the county during the specified period. Other county data include all cases that were sent to MCCO for analysis, but might not represent all unintentional overdose deaths that occurred in those counties. A liquid-chromatography-tandem mass spectrometry–based method, developed and validated by toxicologists at the MCCO Toxicology Laboratory and Department of Chemistry, Wright State University, was used to test for 25 fentanyl analogs, metabolites, and synthetic opioids^§^ in biologic matrices (human blood and urine specimens). 

Toxicologic testing for other substances (heroin, pharmaceutical opioids, benzodiazepines, cocaine, methamphetamine, marijuana, and alcohol) was also conducted. Information on demographic characteristics including age, sex, and race was collected for each decedent. Counties were grouped into the following four urban/rural categories used by the Ohio Department of Health: 1) urban (Montgomery), 2) suburban, 3) rural, non-Appalachian, and 4) Appalachian. The chi-square statistic was used to assess differences among the four county groups in terms of demographic and drug-related characteristics. To examine polydrug patterns, reports of the presence of other fentanyl analogs/metabolites and other drugs were examined for decedents with positive test results for 1) fentanyl, 2) acryl fentanyl, 3) furanyl fentanyl, and 4) carfentanil, one of the most potent fentanyl analogs.

Among the 281 decedents, 122 (43.3%) were from Montgomery County (City of Dayton), a large urban county with a population of approximately 530,000 persons (Table 1). Decedents from four suburban counties, who accounted for 52 (18.5%) unintentional overdose deaths, were primarily from areas that are a part of or adjacent to the Dayton Metro area. Seventy-six (27.0%) decedents were from rural, non-Appalachian counties, primarily from the Southwestern part of the state, and 31 (11.0%) were from the Appalachian counties that are located in the Southern part of the state.

**TABLE 1:** Categories of Ohio counties where unintentional overdose fatalities occurred (N = 281), January–February 2017

County type/name	No. (%) of decedents*
**Urban**	**122 (43.4)**
Montgomery	122 (43.4)
**Suburban**	**52 (18.5)**
Clark	26 (9.3)
Greene	14 (5.0)
Madison	4 (1.4)
Miami	8 (2.8)
**Rural, non-Appalachian**	**76 (27.0)**
Champaign	5 (1.8)
Clinton	6 (2.1)
Darke	7 (2.5)
Fayette	9 (3.2)
Hardin	2 (0.7)
Logan	6 (2.1)
Preble	9 (3.2)
Shelby	9 (3.2)
Warren	15 (5.3)
Wayne	8 (2.8)
**Appalachian**	**31 (11.0)**
Adams	1 (0.3)
Athens	1 (0.3)
Brown	4 (1.4)
Gallia	2 (0.7)
Highland	4 (1.4)
Lawrence	1 (0.3)
Pike	3 (1.1)
Ross	2 (0.7)
Scioto	10 (3.6)
Washington	3 (1.1)
**Total**	**281 (100)**

* For counties other than Montgomery, these numbers represent cases sent to the Montgomery County coroner’s office for an autopsy and might not reflect all overdose deaths in the county.

Males accounted for 181 (64.4%) unintentional overdose deaths, and 257 (91.5%) decedents were white; this proportion was higher in rural (98.7%) and Appalachian (96.8%) counties (p = 0.007) (Table 2). Over half (57.7%) of deaths occurred in persons aged 25–44 years. Approximately 7% of all decedents were not residents of the county where they died, with larger numbers of out of county resident deaths in urban Montgomery County (9.8%).

**TABLE 2:** Demographic and toxicologic characteristics of unintentional overdose fatalities (N = 281), by county type — Ohio, January–February 2017

Characteristic	No. (%)					P-value*
	All cases (N = 281)	Urban (n = 122)	Suburban (n = 52)	Rural (n = 76)	Appalachian (n = 31)	
**Sex**						
Male	**181 (64.4)**	76 (62.3)	34 (65.4)	50 (65.8)	21 (67.7)	0.925
Female	**100 (35.6)**	46 (37.7)	18 (34.6)	26 (34.2)	10 (32.3)	—
**Age group (yrs)**						
<25	**25 (8.9)**	10 (8.2)	3 (5.8)	7 (9.2)	5 (16.1)	0.438
25–34	**82 (29.2)**	32 (26.2)	17 (32.7)	25 (32.9)	8 (25.8)	0.682
35–44	**80 (28.5)**	35 (28.7)	15 (28.8)	21 (27.6)	9 (29.0)	0.998
45–54	**54 (19.2)**	28 (23.0)	6 (11.5)	14 (18.4)	6 (19.4)	0.376
≥55	**40 (14.2)**	17 (13.9)	11 (21.2)	9 (11.8)	3 (9.7)	0.402
**Race**						
White, non-Hispanic	**257 (91.5)**	109 (89.3)	43 (82.7)	75 (98.7)	30 (96.8)	0.007
African American or Other	**24 (8.7)**	13 (10.6)	7 (17.3)	1 (1.3)	1 (3.2)	—
**Residence status**						
Out of county residents	**19 (6.8)**	12 (9.8)	—	6 (7.9)	1 (3.2)	—
**Synthetic opioids/Fentanyl analogs/Metabolites**						
Fentanyl	**253 (90.0)**	113 (92.6)	46 (88.5)	67 (88.2)	27 (87.1)	0.648
Norfentanyl	**157 (55.9)**	72 (59.0)	26 (50.0)	44 (57.9)	15 (48.4)	0.563
Acryl fentanyl	**136 (48.4)**	61 (50.0)	31 (59.6)	35 (46.1)	9 (29.0)	0.056
Despropionylfentanyl (4-ANPP)	**118 (42.0)**	55 (45.1)	29 (55.8)	26 (34.2)	8 (25.6)	0.021
Despropionyl para-Fluorofentanyl	**1 (0.4)**	—	—	—	1 (3.2)	—
Furanyl Fentanyl	**87 (31.0)**	45 (36.9)	19 (36.5)	17 (22.4)	6 (19.4)	0.062
Furanyl Norfentanyl	**2 (0.7)**	1 (0.8)	—	1 (1.3)	—	—
Carfentanil	**21 (7.5)**	11 (9.0)	3 (5.8)	6 (7.9)	1 (3.2)	—
Acetyl fentanyl	**4 (1.4)**	2 (1.6)	—	1 (1.3)	1 (3.2)	—
Butyryl/Isobutyrylfentanyl	**4 (1.4)**	1 (0.8)	3 (5.8)	—	—	—
Butyryl norfentanyl	**2 (0.7)**	—	2 (3.8)	—	—	—
Fluorobutyryl/Fluoroisobutyrylfentanyl	**3 (1.1)**	—	1 (1.9)	1 (1.3)	1 (3.2)	—
U-47700^†^	**2 (0.7)**	1 (0.8)	1 (1.9)	—	—	—
Any type of fentanyl/analog	**259 (92.2)**	117 (95.9)	47 (90.4)	68 (89.5)	27 (87.1)	0.216
**Other opioids**						
Heroin^§^	**16 (5.7)**	3 (2.5)	2 (3.8)	3 (3.9)	8 (25.8)	<0.001
Heroin, no type of fentanyl/analog	**4 (1.4)**	—	1 (1.9)	2 (2.6)	1 (3.2)	–
Any pharmaceutical opioid	**64 (22.8)**	26 (21.3)	11 (21.2)	18 (23.7)	9 (29.0)	0.813
Hydrocodone	**15 (5.3)**	5 (4.1)	3 (5.8)	5 (6.6)	2 (6.5)	0.874
Oxycodone	**30 (10.7)**	11 (9.0)	4 (7.7)	9 (11.8)	6 (19.4)	0.335
Oxymorphone	**3 (1.1)**	—	—	1 (1.3)	2 (6.5)	—
Methadone	**10 (3.6)**	7 (5.7)	1 (1.9)	1 (1.3)	1 (3.2)	—
Morphine^¶^	**9 (3.2)**	5 (4.1)	2 (3.8)	0	2 (6.5)	—
Buprenorphine**	**1 (0.7)**	—	—	1 (1.3)	—	—
Loperamide**	**1 (0.7)**	—	—	1 (1.3)	—	—
Tramadol	**10 (3.5)**	4 (3.3)	3 (5.8)	3 (3.9)	—	—
**Other drugs**						
Cocaine	**86 (30.6)**	46 (37.7)	22 (42.3)	17 (22.4)	1 (3.2)	<0.001
Methamphetamine	**33 (11.7)**	14 (11.5)	5 (9.6)	9 (11.8)	5 (16.1)	0.847
Marijuana	**99 (35.2)**	43 (35.2)	20 (38.5)	23 (30.3)	13 (41.9)	0.644
Alcohol	**57 (20.3)**	25 (20.5)	11 (21.2)	16 (21.1)	5 (16.1)	0.943
Benzodiazepines (any)	**75 (26.6)**	37 (30.3)	12 (23.1)	20 (26.0)	6 (19.4)	0.562
Gabapentin**	**11 (3.9)**	2 (1.6)	2 (3.8)	4 (5.2)	3 (9.7)	—

* Chi-square p*-*value for comparison across four county groups; p<0.05 is considered statistically significant.

^†^ Synthetic opioid not structurally related to fentanyl.

^§^ Cases that tested positive for 6-MAM and/or were identified by the coroner as heroin-related.

^¶^ Only cases that tested for morphine but not 6-MAM, and were not identified by the coroner as heroin-related.

** Not all cases were tested for buprenorphine, loperamide, or gabapentin. Testing was performed only when evidence of misuse was present.

Overall, 253 (90.0%), 136 (48.4%), and 87 (31.0%) decedents tested positive for fentanyl, acryl fentanyl, and furanyl fentanyl, respectively (Table 2). The proportions of decedents that were positive for acryl fentanyl and furanyl fentanyl were lower in Appalachian counties (29.0% and 19.4%, respectively), although these differences were not statistically significant. There were statistically significantly more decedents in urban and suburban counties that tested positive for despropionylfentanyl (4-ANPP) (45.1% and 55.8%, respectively) than in rural (34.2%) and Appalachian (25.6%) counties (p = 0.021).

Only 16 (5.7%) of all 281 decedents tested positive for heroin, with a significantly higher proportion in Appalachian counties (25.8%) than in urban (2.5%), suburban (3.8%) or rural non-Appalachian counties (3.9%). Among all 16 heroin-positive cases, 12 also tested positive for IMF. Overall, 64 (22.8%) decedents tested positive for pharmaceutical opioids, 75 (26.6%) for benzodiazepines, and 86 (30.6%) for cocaine; a higher percentage of decedents who tested positive for cocaine died in urban (37.7%) and suburban (42.3%) counties than in rural (22.4%) or Appalachian (3.2%) counties (p<0.001) (Table 2).

Over half (53.8%) of specimens from fentanyl-positive decedents also tested positive for acryl fentanyl, and approximately one third (34.0%) for furanyl fentanyl (Table 3). Approximately 62% of fentanyl-positive decedents did not test positive for norfentanyl. All specimens from acryl fentanyl deaths also tested positive for fentanyl, and 39.7% tested positive for furanyl fentanyl. Approximately 99% of furanyl fentanyl deaths tested positive for fentanyl, 62.1% for acryl fentanyl, and 86.2% for 4-ANPP.

**TABLE 3:** Presence of other drugs in fentanyl-, acryl fentanyl–, furanyl fentanyl– and carfentanil-positive unintentional overdose deaths (N = 281) — Ohio, January–February 2017

Type of drug/metabolite	No. (%)			
	Fentanyl (n = 253)	Acryl fentanyl (n = 136)	Furanyl fentanyl (n = 87)	Carfentanil (n = 21)
Fentanyl	NA	136 (100)	86 (98.9)	15 (71.4)
Acryl fentanyl	136 (53.8)	NA	54 (62.1)	5 (23.8)
Furanyl fentanyl	86 (34.0)	54 (39.7)	NA	8 (38.1)
Carfentanil	15 (5.9)	5 (3.7)	8 (9.2)	NA
Norfentanyl	157 (62.1)	80 (58.8)	54 (62.1)	10 (47.6)
Despropionylfentanyl (4-ANPP)	117 (46.2)	72 (52.9)	75 (86.2)	11 (52.4)
Despropionyl para-fluorofentanyl	1 (0.4)	1 (0.7)	—	—
Furanyl norfentanyl	2 (0.8)	1 (0.7)	2 (2.3)	1 (4.8)
Acetyl fentanyl	4 (1.6)	3 (2.2)	2 (2.3)	—
Butyryl/Isobutyrylfentanyl	4 (1.6)	1 (0.7)	1 (1.1)	—
Butyryl norfentanyl	2 (0.8)	—	1 (1.1)	—
Fluorobutyryl/Fluoroisobutyrylfentanyl	3 (1.2)	1 (0.7)	—	—
U-47700	2 (0.8)	1 (0.7)	2 (2.3)	—
**Other drugs**				
Heroin	12 (4.7)	3 (2.2)	3 (3.4)	—
Pharmaceutical opioids (any)	51 (20.2)	265(18.4)	18 (20.7)	5 (23.8)
Benzodiazepines (any)	65 (25.7)	35 (24.6)	24 (27.6)	9 (42.9)
Cocaine	78 (30.8)	41 (30.1)	29 (33.3)	9 (42.9)
Methamphetamine	32 (12.6)	13 (9.6)	10 (11.5)	2 (9.5)
Marijuana	91 (36.0)	44 (32.4)	38 (43.7)	12 (57.1)
Alcohol	46 (18.2)	19 (14.0)	15 (17.2)	2 (9.5)

**Abbreviation:** NA = not applicable.

Twenty-one decedents (including 11 [52%] in Montgomery County) tested positive for carfentanil. Among these, 15 (71.4%) decedents also tested positive for fentanyl, five (23.8%) for acryl fentanyl, and eight (38.1%) for furanyl fentanyl. Many of the carfentanil decedents tested positive for other central nervous system depressants, such as pharmaceutical opioids (23.8%) and benzodiazepines (42.9%). Approximately 30% of fentanyl, acryl fentanyl, and furanyl fentanyl cases tested positive for cocaine. Among carfentanil cases, approximately 40% were positive for cocaine (Table 3).

## Discussion

Evidence from the toxicologic analyses of unintentional overdose deaths in Ohio from the beginning of 2017 indicate the increasing and substantial role of IMFs, and the declining presence of heroin and pharmaceutical opioids in overdose fatalities, compared with 2014–2016 data from Ohio and other states (*3*–*5*). Approximately 90% of unintentional overdose deaths in 24 Ohio counties that occurred during January and February 2017 involved fentanyl, fentanyl analogs, or both. Approximately 32% of fentanyl-positive decedents did not test positive for norfentanyl, a major metabolite for fentanyl, suggesting a very rapid death (*6*). Twenty-one decedents tested positive for carfentanil, a highly toxic IMF compound (approximately 10,000 times more potent than morphine), which is frequently used in veterinary medicine for sedation of large animals. Approximately one third of unintentional overdose deaths that tested positive for IMF also tested positive for cocaine. It is not known whether these data indicate a pattern of intended polydrug use or if cocaine and IMF mixtures were sold to unsuspecting illicit opioid or cocaine users.

The study documents the high numbers of acryl fentanyl– and furanyl fentanyl–associated deaths among unintentional overdose fatalities in the United States. Acryl fentanyl is more potent than fentanyl (*7*); in 2016, there were reports of furanyl fentanyl–related overdoses in Canada caused by smoking contaminated cocaine (*8*). These drugs are commonly advertised on cryptomarkets, which are commercial web-based marketplaces for transactions involving drugs and other illicit goods that provide anonymity to both buyers and sellers via their location on the “Dark” web (internet content that requires specific software or authorization to access) and use of cryptocurrencies (e.g., bitcoin) for payment. Nearly half of fentanyl positive cases and approximately 90% of furanyl fentanyl positives tested positive for 4-ANPP. 4-ANPP is used as a precursor for the manufacture of fentanyl-type drugs; it is also an impurity found in fentanyl preparations and is a metabolite of fentanyl and furanyl fentanyl (*9*).

The findings in this report are subject to at least four limitations. First, for counties other than Montgomery, unintentional overdose numbers represent cases sent to MCCO for an autopsy and might not reflect all overdose deaths in that county. Further, it is not known whether there are systemic differences across counties (other than Montgomery County) regarding the types of cases sent to MCCO for testing. Second, toxicology reports cannot distinguish between pharmaceutical and illicitly manufactured fentanyl, although previous reports indicate that the majority of fentanyl linked to fatal unintentional overdoses in the country is suspected to be IMF (*10*). Third, toxicology data on decedents testing positive for multiple drugs cannot determine if the decedent knowingly or unknowingly used combinations of different drugs. Finally, data were obtained from 24 Ohio counties, and findings might not be generalizable to the entire state.

Overall, IMFs are appearing in combination with other fentanyl analogs, and co-occurrence of other drugs is common. The high percentage of overdose fatalities testing positive for combinations of IMFs might indicate that available street drugs include mixtures of different types of IMFs or that persons use drugs obtained from multiple sources, with different toxicologic profiles. Expansion of access to evidence-based treatment is an important strategy for preventing fentanyl-related overdoses (*3*). These findings highlight the urgent need to make IMF testing a part of standard toxicology panels for biological specimens used by substance abuse treatment centers, criminal justice institutions, and medical providers. Implementation of harm reduction initiatives could also help reduce the adverse consequences of IMF use (*3*,*5*). Because multiple naloxone doses are often required to reverse overdoses from IMFs (*5*), assuring that sufficient supplies are provided to first responders and distributed through community overdose prevention programs can mitigate the effects of opioid overdoses.

**Summary**

**What is already known about this topic?**

Illicitly manufactured fentanyl has become a significant contributor to unintentional overdose deaths in the United States.

**What is added by this report?**

Approximately 90% of unintentional overdose deaths examined in 24 Ohio counties that occurred during January–February 2017 involved fentanyl, fentanyl analogs, or both, whereas heroin was identified in the minority (6%) of cases, with somewhat higher prevalence in Appalachian counties. Fentanyl is commonly appearing in combination with other analogs.

**What are the implications for public health practice?**

These findings highlight the urgent need to make illicitly manufactured fentanyl testing a part of standard toxicology panels for biological specimens. Because multiple naloxone doses are often required to reverse overdoses from illicitly manufactured fentanyl, assuring that sufficient supplies are provided to first responders and distributed through community overdose prevention programs can mitigate the effects of opioid overdoses.

## Footnotes

*
http://www.odh.ohio.gov/-/media/ODH/ASSETS/Files/health/injury-prevention/2015-Overdose-Data/2015-Ohio-Drug-Overdose-Data-Report-FINAL.pdf .

† Selected data include all cases analyzed during January 1–February 28, 2017, from Montgomery County and all other nonurban counties that submitted cases for analysis to the Montgomery County Coroner's Office Toxicology Laboratory.

§ The 25 compounds are 1) 1-3-methylfentanyl; 2) 4-ANPP; 3) acetyl fentanyl; 4) acetyl fentanyl 4-methylphenethyl; 5) acryl fentanyl; 6) alfentanil; 7) beta-hydroxythiofentanyl; 8, 9) butyryl fentanyl/isobutyryl fentanyl; 10) butyryl norfentanyl; 11) carfentanil; 12) despropionyl para-fluorofentanyl; 13) fentanyl; 14) furanyl fentanyl; 15) furanyl norfentanyl; 16) norfentanyl; 17, 18) fluorobutyryl/fluoroisobutyrylfentanyl; 19) para-methoxyfentanyl; 20) remifentanil; 21) remifentanil metabolite; 22) sufentanil; 23) valeryl fentanyl; 24) AH7921; and 25) U-47700. The latter two are synthetic opioids not structurally related to fentanyl.
