# *Errata*: Vol. 66 No. 34

**DOI:** 10.15585/mmwr.mm6638a8

**Published:** 2017-09-29

In the report, “Overdose Deaths Related to Fentanyl and Its Analogs — Ohio, January–February 2017,” on page 904, the sixth sentence of the first paragraph should have read “The Wright State University and the Montgomery County Coroner’s Office/Miami Valley Regional Crime Laboratory (MCCO/MVRCL) collaborated on a National Institutes of Health study of fentanyl analogs and metabolites and other drugs identified in 281 unintentional overdose fatalities in **25** Ohio counties during January–February 2017.”

On page 904 the second sentence of the second paragraph should have read “Data from 281 unintentional overdose fatalities that occurred in Montgomery County and **24** additional counties^†^ during January and February 2017, were analyzed by the MCCO Toxicology laboratory, and had assigned causes of death as of May 8, 2017, were included in this study.”

On page 905, under “What is added by this report?” the first sentence should have read “Approximately 90% of unintentional overdose deaths examined in **25** Ohio counties that occurred during January–February 2017 involved fentanyl, fentanyl analogs, or both, whereas heroin was identified in the minority (6%) of cases, with somewhat higher prevalence in Appalachian counties.”

On page 907, the second sentence of the first paragraph should have read “Approximately 90% of unintentional overdose deaths in **25** Ohio counties that occurred during January and February 2017 involved fentanyl, fentanyl analogs, or both.

On page 907, the last sentence of the third paragraph should have read “Finally, data were obtained from **25** Ohio counties, and findings might not be generalizable to the entire state.”

